# rtMEG: A Real-Time Software Interface for Magnetoencephalography

**DOI:** 10.1155/2011/327953

**Published:** 2011-05-17

**Authors:** Gustavo Sudre, Lauri Parkkonen, Elizabeth Bock, Sylvain Baillet, Wei Wang, Douglas J. Weber

**Affiliations:** ^1^Program in Neural Computation, Carnegie Mellon University, Pittsburgh, PA 15213, USA; ^2^Brain Research Unit, Low Temperature Laboratory, Aalto University School of Science, 00076 Espoo, Finland; ^3^Department of Neurology, Froedtert & The Medical College of Wisconsin, Milwaukee, WI 53226, USA; ^4^Departments of Neurology and Biophysics, Froedtert & The Medical College of Wisconsin, Milwaukee, WI 53226, USA; ^5^Department of Physical Medicine and Rehabilitation, University of Pittsburgh, Pittsburgh, PA 15260, USA

## Abstract

To date, the majority of studies using magnetoencephalography (MEG) rely on off-line analysis of the spatiotemporal properties of brain activity. Real-time MEG feedback could potentially benefit multiple areas of basic and clinical research: brain-machine interfaces, neurofeedback rehabilitation of stroke and spinal cord injury, and new adaptive paradigm designs, among others. We have developed a software interface to stream MEG signals in real time from the 306-channel Elekta Neuromag MEG system to an external workstation. The signals can be accessed with a minimal delay (≤45 ms) when data are sampled at 1000 Hz, which is sufficient for most real-time studies. We also show here that real-time source imaging is possible by demonstrating real-time monitoring and feedback of alpha-band power fluctuations over parieto-occipital and frontal areas. The interface is made available to the academic community as an open-source resource.

## 1. Introduction

 Off-line analysis of magnetoencephalography (MEG) data has been applied to a wide spectrum of basic and clinical neuroscience questions (see, e.g., [1, 2]). The ability to process and analyze MEG data in real time would potentially open new opportunities for neuroscientific research and innovative clinical applications. For example, adaptive paradigms (or optimal experiment designs [3, 4]) would benefit from the possibility of capturing MEG measurements in real time, for example, to select the most efficient stimulus type, or to determine which stimulus classes necessitate the collection of more repetitions in order to increase classification accuracy in the context of a cognitive-state decoding task. Moreover, real-time neurofeedback could be used to train subjects to modulate some specific spatial and dynamic features of their neural activity in the context of brain-machine interface (BMI) applications. From a clinical standpoint, neurofeedback training may help promote neuroplasticity to reinforce spared corticospinal pathways after stroke or spinal cord injury [5, 6]. 

While systems that use real-time feedback with different MEG machines have been previously described [7–11], this work presents a software interface (“rtMEG”) designed to acquire signals from an Elekta Neuromag device in real time. It provides the following additional features with respect to the software that was described previously [12].

- This version of the software interface is more robust and is better integrated into the standard MEG acquisition system. For example, it performs data acquisition using the set of parameters specified through the regular acquisition software interface. Furthermore, data are streamed with proper channel calibration and ordering. In the near future, users will also have the option to stream the data with online signal-space projection (SSP) [13] noise reduction being applied, while currently this transformation should be performed on the client workstation.
- The rtMEG interface now writes data to the Fieldtrip buffer [14], instead of being integrated into the BCI2000 pipeline. The Fieldtrip buffer consists of an open-source server program that runs continuously, providing a shared memory buffer to which rtMEG writes the data. While it was possible to stream the data out of BCI2000 in the previous implementation, that software was still required to run rtMEG. With the current implementation, researchers have the freedom to use whatever solutions they favor by running the Fieldtrip buffer implemented within rtMEG and using the code freely available online [15] to read from the buffer. Moreover, researchers have the option to work with any of the Fieldtrip tools used for off-line analysis in an on-line setting. Another advantage of using the Fieldtrip buffer is the independence on the operating system. While the buffer has been implemented within rtMEG, the user still has the option to run it externally under Windows, MacOS, and Linux/Unix using the software provided by the Fieldtrip developers (in contrast, BCI2000 is mostly run on Windows). Finally, the Fieldtrip buffer provides the flexibility to interact with other commonly used software packages (BCI2000 [16], Brainstream [17], among others), and because the code to read from the buffer is freely available online [15], researchers can easily integrate it to their own custom solutions.
- rtMEG can be modified and compiled using open source software.

It is important to note that although the rtMEG interface does not depend on BCI2000 anymore [12], it is still able to interact with the latter. Indeed, BCI2000 can read from the Fieldtrip buffer either by using the Fieldtrip buffer source module or the Remote Data Access streaming interface.

We describe the system setup and the tests that were performed to assess the delay in accessing the data stream. We then show results regarding acquisition delays and illustrate the technique with real-time source estimation in a neurofeedback experiment. We conclude with a discussion of several scenarios where we foresee that the rtMEG interface may prove useful.

## 2. Methods

**Figure 1:**
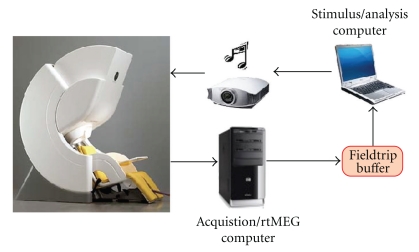
Illustration of a typical setup with rtMEG. The acquisition computer controls the acquisition, stores the data, and runs the rtMEG interface. Another computer drives the experimental paradigm by providing stimuli to the subject and sending trigger events that eventually go to the MEG data file. The rtMEG interface writes data to the Fieldtrip buffer, which can be run by rtMEG or by any other computer in the network. The application(s) reading from this buffer can run on the stimulus computer or on any other computer connected in the network.

 The interface was developed to function in conjunction with the standard MEG acquisition, without affecting the normal workflow. In a typical scenario (Figure 1), a dedicated computer runs the main acquisition software and saves the acquired data on the MEG filesystem. The rtMEG interface runs on this acquisition workstation and operates in parallel with the standard acquisition software.

In a typical experimental setting, a separate computer controls stimulus delivery to the subject. Stimuli may comprise multiple categories (auditory, visual, etc.). For synchronization, the stimulus computer sends event-related trigger pulses through the parallel port to mark the onsets of stimuli in the recorded files.

rtMEG writes data to a Fieldtrip buffer that can be either run by rtMEG itself or hosted by any other computer located in the same network as the acquisition computer (e.g., the stimulus computer). This buffer can then be read using Matlab [18] (with Fieldtrip scripts) or another preferred solution (see the code openly available on the Fieldtrip website [15]). Similarly, the computer reading from the buffer can be the same as the computer hosting the buffer, or any other computer in the same network.

### 2.1. Details of Implementation and Distribution

 In the usual setup, each Digital Signal Processor (DSP) unit manages 12 channels in the MEG machine, and packets comprising 28 samples per channel are sent by each DSP to the real-time computer, which reorders and synchronizes the data and attaches metainformation, such as calibration coefficients and sampling rate, to them. The acquisition computer, which also runs rtMEG, receives the data from the real-time computer. When using typical sampling rates (<1.5 kHz), the data are sent to the acquisition computer in chucks of about 1 s, which corresponds to a considerable and often unacceptable delay for any real-time application. However, rtMEG can optionally reduce the size of the chunk, down to a lower bound of 28 samples, by reconfiguring the real-time computer and thereby substantially diminishing the average transit delay of the data.

The data received from the real-time computer are then stored in a local shared memory buffer that is used by different Neuromag programs, such as the on-line visualization. rtMEG taps into this local buffer, reads the data, and writes them to a Fieldtrip buffer, which can then be easily read by several different clients using an open-source format. This Fieldtrip buffer can be run by rtMEG itself in a separate thread, or by a separate computer in the network.

rtMEG was written in C, and all network communication is done using TCP/IP. The source code is made available to the research community under Gnu Public License (GPL) and stored in the Fieldtrip source control repository. Documentation [19] has been written in the Fieldtrip Wiki. Binary files for HP-UX and Linux platforms have also been provided for the users' convenience.

### 2.2. Assessing Delays to Data Access

**Figure 2:**
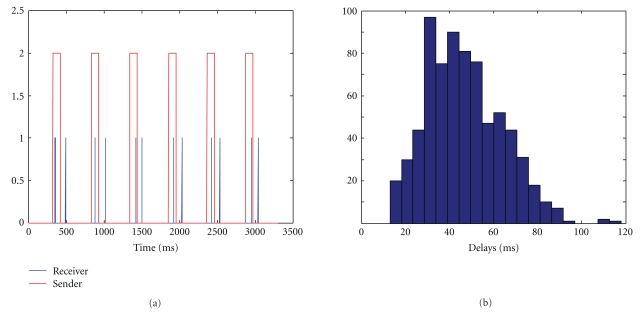
Measuring the data-access delay in real time. (a) Red pulses correspond to the signal created by the acquisition workstation; blue traces show the time when the computer reading from the Fieldtrip buffer detected the pulse and sent an acknowledgment pulse back to the acquisition computer. The delay was measured by calculating the time difference between all vertical red bars and the corresponding blue bars. (b) Histogram of the observed delays during a 5-minute measurement with buffer size 29.

 Real-time MEG applications often rely on minimal system delays, and the rtMEG interface needs to be carefully assessed in this respect. We measured the delay associated to complete feedback loop as follows. We recorded 306 MEG channels and 3 stimulus channels at 1 kHz. These data were written to a Fieldtrip buffer implemented inside rtMEG and then read over the network by a separate Linux computer. Data were written to and read from the buffer every 29 samples. The acquisition software was set to generate a pulse (square wave) in one of the stimulus trigger channels every 500 ms (rise from zero level to value “2”, hold on for 100 ms, and then return to zero). The Linux computer ran a simple C program that was designed to write a logical “1” to the parallel port every time a change was detected on the trigger channel, and a logical “0” otherwise. The parallel port was mapped on to a different stimulus trigger channel in the data. Because the MEG system acquires all signals synchronously, this form of testing using the trigger input is indicative of the data-access delays in the system. Delays were measured as the time difference between the occurrences of “1”—when the Linux computer responded to a change in the trigger—and “2”—marking the actual occurrences of the change—in the data; see Figure 2(a).

### 2.3. Real-Time Feedback and MEG Source Imaging

 The primary goal of real-time operations is to provide the subject with a measure of his/her brain activity. To prove and evaluate this technical concept, an experiment was designed to report on variations of ongoing regional brain activity related to behavior. This objective was challenging because it implied that both (1) data acquisition and formatting, and (2) source modeling of ongoing brain activity, were achievable in real time. To our knowledge, this latter feature had not been demonstrated with EEG or MEG so far. Here, we designed a simple paradigm in which the subject was alternating 20-s segments of rest with his eyes either closed or open. An auditory cue was provided to the subject to let him know when to open or close his eyes. It is a very well-documented and robust phenomenon that the amplitude of alpha (8–13 Hz) oscillations is stronger over the dorsal parietal and posterior occipital brain regions with the eyes being closed versus open.

Real-time estimation of ongoing alpha power was performed over a set of cortical regions of interest (ROIs) that were predefined from the individual brain anatomy of one subject. The ROIs covered the dorsal parietal and posterior occipital (PO) cortex and were delineated using BrainStorm [20] (Figure  4(a)). An additional ROI was defined over the anterior and dorsolateral prefrontal cortex, for comparison with the levels of alpha power changes observed in the parieto-occipital region. The cortical surface was obtained from the T1-weighted volume MRI (1.5 T, SPGR sequence, voxel size: 0.9 × 0.9 × 1.5 mm^3^; field of view: 240 × 240 mm) using BrainVISA [21]. MEG data acquisition and analysis were performed at Froedtert & the Medical College of Wisconsin (Milwaukee, USA) using a 306-channel Elekta Neuromag MEG system. 

The entire recording session lasted 10 minutes and consisted of a short 10-s baseline run, followed by 3 runs of 130 seconds each. The subject's head position was measured at the beginning of each run by the software provided with the MEG system. The head location from the short baseline run was used by the forward head modeling and inverse source modeling steps necessary to access cortical source estimates from ongoing MEG data. Both steps were completed in approximately 2 minutes using BrainStorm after the baseline run was acquired. Head modeling was performed using the overlapping-sphere analytic approach [22]. The linear imaging kernel from BrainStorm's weighted and cortically constrained minimum-norm estimate (WMNE) [23] was subsequently obtained and stored in memory. Because the WMNE is a linear, stationary source estimation approach, source signals can be readily accessed from each real-time buffer data by simply completing the matrix multiplication of the imaging kernel with either the sensor data time series or Fourier coefficients. In our study, this was further reduced to the extraction of the elementary sources within the targeted ROIs, which amounted to about 750 current dipoles.

For each 500-ms segment, the power in the alpha range across the PO ROI was computed from the Fourier coefficients of each of the 750 elementary sources. These were obtained by applying the imaging kernel to the fast-Fourier transform (FFT) coefficients of the running segment of sensor data. The power in each ROI therefore consisted of the sum of the magnitude of the resulting Fourier coefficients in the 8–13 Hz range across the entire set of elementary sources forming the ROI. The cumulative time taken to perform this operation—magnitude of the product of a 750 × 306 imaging kernel by 306 × 1,000 Fourier coefficients of MEG sensor data—was about 100 ms on a conventional workstation running Matlab.

The overall benefits of the imaging kernel and Fourier-domain approach were that the time-consuming steps of the forward and inverse modeling were performed offline. The downside was the suboptimal accuracy of these models due to cumulative head movements during the session. These movements were evaluated from the measurements of the head positions collected at the beginning of each of the 3 feedback runs.

**Figure 3:**
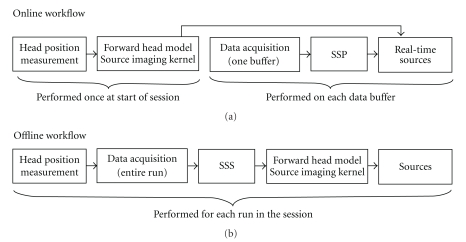
Workflows of real-time data collection and processing using rtMEG (a) and of the optimal, off-line processing chain (b). For real-time data analysis, the forward head model and inverse imaging kernel were precomputed and applied online on all subsequent recordings. This differs from the optimal off-line pipeline, where forward and inverse modeling is completed for each individual run. In addition, interference suppression was performed in the client workstation using SSP on each data buffer during real-time data collection and analysis, whereas SSS was used over the entire duration of each individual run during the off-line analysis.

State-of-the-art MEG acquisition may also include active denoising techniques, requiring both on-line and off-line processing steps to be performed. In the case of the MEG installation used for this study, the standard data acquisition pipeline consists of (1) the on-line application of signal-space projection (SSP) to compensate for the spatial pattern of some environmental interference sources and (2) the off-line application of the signal-space separation (SSS) technique [24], to fully benefit from the latest generation of single-layer magnetically shielded rooms.  Figure 3 details the approach we used in the present study to assess the deviations of the outcome of the real-time data acquisition and source analysis from the conventional, optimal pipeline that is only accessible offline.

**Figure 4:**
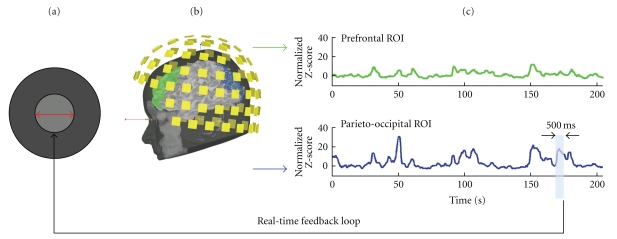
Real-time visual feedback on the power of alpha oscillations in brain regions of interest. (a) The subject was provided with a visual gauge of the real-time level of alpha power within the parieto-occipital (PO) region of interest shown in blue in (b). The radius of the light-grey disk in (a) evolved every 500 ms and increased as alpha power decreased during the eyes-open segment of the experimental run. The static, dark-grey disk was an incentive target for the subject. Its radius was indexed to 2 times the average PO alpha power captured during the baseline run acquired at the beginning of the session. (c) shows the ongoing, respective levels of alpha power variation in the two ROIs: prefontal (in green in b) and PO (in blue in b).

Real-time visual feedback on the level of alpha power within the target ROIs was provided to the subject after the processing of each 500-ms data segment by the stimulus computer that was hosting the FieldTrip buffer (see Figure 4(a)). These measurements of brain activity were saved to a disk file and converted to a visual display that was provided to the subject via a video projection system (60-Hz refresh rate). During the segments with eyes open, the subject was instructed to try to maximize the level of the visual gauge, which was indexed to the inverse of the power of alpha oscillation in the targeted ROIs (Figures 4(b) and 4(c)).

## 3. Results

 The following sections describe the results obtained while measuring the data-access delays introduced by the rtMEG interface to the data stream, and the results observed while providing real-time feedback of alpha-band power modulation.

### 3.1. Delay Measurements

The average delay to access the data was measured to be 44 ± 17 ms, and it was insensitive to the number of channels being simultaneously transmitted over the network. No changes were noticed after the system continuously collected data for several minutes. A histogram of the observed delays during a representative measurement is shown in Figure 2(b). The variability of the results is attributed to the asynchrony between the change in the trigger channel and the boundaries of the 29-sample buffer. Hence, the theoretical distribution should show the mean data-access delay time ±29 ms (1000-Hz sampling rate). However, the program that read the data from the buffer was designed to run in an infinite loop, and whenever there was no new data in the buffer since the last read action, it paused for a predetermined amount of time. This sleep time is responsible for the subtle dissimilarities between the theoretical distribution and the histogram shown in Figure 2(b). The overall results show that the interface introduced only modest delays to the measured signal, which are likely to be short enough for most real-time MEG applications. The distribution of delays was also consistent over time.

The delay values reported here are slightly higher than what was reported before [12], which is justified because of the different ways in which the two implementations access the MEG data. While the previous implementation collected the data directly from the DSPs, the current implementation reads the data from the local buffer in the acquisition computer. Moreover, the previous implementation did not sort and calibrate the channels as is now done by the real-time computer. The current implementation is preferred because it provides a more intuitive and robust interface to the user without repeating processing steps that are already reliably implemented in the real-time computer while still keeping the data-access delay at an acceptable level.

It is important to reiterate that this experiment measured the delay to access the data; more complex real-time processing will likely increase the overall system delay. 

### 3.2. Real-Time Source Imaging

**Figure 5:**
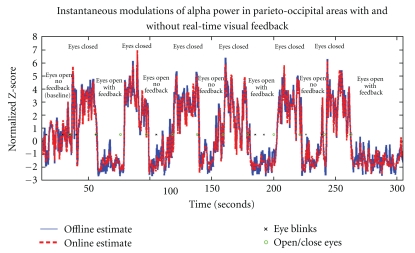
Comparison of off-line (in blue) versus on-line (in red) estimates of alpha power modulations over the PO ROI. The traces of the time-resolved power estimates are plotted over time and were standardized over the 20-s baseline period immediately preceding the first eyes-closed segment. As expected, eye blinks (marked with an “x”) were detected from the EOG channel during the segments where the subject had his eyes open. The transitions from open to closed eyes are marked at the time the subject was given an auditory stimulus (marked with an “o”) every 20 s. In this particular run (300 s), the root-mean-square (RMS) difference between the online and offline time series reached 29.2%. Both time series indicate lower and more sustained decreases of alpha power in the PO region with respect to baseline, when feedback was provided to the subject than when no feedback was shown.

Both the on-line and off-line source analyses revealed modulations of oscillatory alpha power within the PO region (Figure 5). These measures were standardized (Z-score) with respect to a baseline data segment of reference obtained in the first 20 seconds of each feedback run (subject resting with eyes open, fixating at a crosshair on the screen). As shown in Figure 5, excursions under the baseline alpha levels were stronger and more sustained during the segments with eyes open and feedback than when no feedback was provided, indicating an encouraging trend that feedback indeed drove the subject towards lower alpha levels than during baseline, and during segments where no feedback was present. 

**Figure 6:**
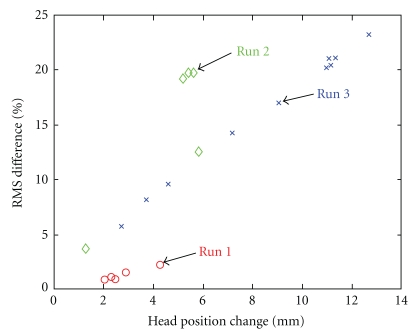
RMS differences in the alpha power estimates resulting from changes in the head position across three different sessions, each session consisting of multiple runs of 130-s duration each. The differences in head position are with respect to the one captured during an initial baseline run acquired at the beginning of the first session. The on-line calculations of alpha power modulations using the reference head position were compared to those obtained offline but with optimal noise attenuation and forward head and inverse source modeling. Colors represent different sessions, and each marker represents a run within a session.

Comparison of the off-line and on-line estimates of alpha power modulations in the PO regions qualitatively demonstrated that the data were not altered or significantly delayed by the transfer from the acquisition to the analysis workstation, and/or by the optimal denoising techniques applied and more accurate head/source models (Figure 5). The discrepancies observed—reaching up to 24.5% RMS error as in Figure 5—showed strong dependence on the fluctuations in the subject's head position over time, reaching a maximum of 12.5 mm (see Figure 6). 

## 4. Conclusions

The analysis of MEG signals in real time opens up new possibilities for the study of brain function. Potential applications include the following.

- *Basic Research.* Real-time visualization of MEG data in source space (on the brain surface) for quality assurance and rapid interpretation of the measurement. Dynamic and adaptive paradigms where subject's brain state could be a condition to stimulus delivery.
- *Brain-Machine Interfaces.* Our previous off-line MEG studies have shown that we can decode intended movement direction from MEG signals and accurately localize cortical areas representing such information for real-time BMI operation [25]. With the real-time capability, it will be beneficial to use MEG as a presurgical tool to localize the optimal placement site for an ECoG grid for obtaining real-time BMI control. Furthermore, researchers may test various neural processing, decoding, and user training paradigms “on the fly” within a single MEG session.
- *Clinical.* Real-time neurofeedback training can be used to promote neuroplasticity [5, 6]. Through the operation of an rtMEG-BMI system, users can learn to voluntarily modulate or change their brain activity [7, 8], inducing neuroplasticity for recovery of motor function or to improve control of neuroprosthetic devices.

This paper described a software solution that enables easy real-time access to the MEG signals from any computer connected to the local network. We demonstrated that the delay to access the data by this software was minimal, and that the access mechanism easily lends itself to real-time source modeling.
